# Evaluation of an application for investments in cataract surgery, Ethiopia

**DOI:** 10.2471/BLT.25.293592

**Published:** 2026-04-29

**Authors:** Dominque Geoffrion, Noelle Whitestone, Jennifer L Patnaik, Samson Tesfaye, Dawit Seyum, Nigusie Fetene, Enock Nsokolo, Lucia Nadaf, Ruth Nswana, Alemayehu Sisay, David H Cherwek, Serge Resnikoff, Phylis Moonga, Jonathan Ncheengamwa, Nathan Congdon

**Affiliations:** a Department of Ophthalmology, University of Montreal, Montreal, Quebec, Canada.; b Orbis International, 8th Floor, 52 Vanderbilt Ave, New York, NY 10017, United States of America (USA).; c Department of Ophthalmology, University of Colorado School of Medicine, Aurora, Colorado, USA.; d Orbis Ethiopia, Addis Ababa, Ethiopia.; e Orbis Zambia, Lusaka, Zambia.; f School of Optometry and Vision Science, Faculty of Medicine, University of New South Wales, Sydney, Australia.; g Ministry of Health, Lusaka, Zambia.; h Konkola Mine Hospital, Chililabombwe, Zambia.

## Abstract

**Problem:**

The World Health Organization recommends a benchmark for uncorrected visual acuity of ≥ 6/12 in 80% of patients at 4 to 6 weeks following cataract surgery. In low-resource countries, reaching this benchmark is challenging and assessing progress is difficult.

**Approach:**

This intervention involved modest investments recommended by the Better Operative Outcome Software Tool (BOOST) application (app). The investments targeted specific causes of poor cataract surgery results at hospitals in Ethiopia to improve vision outcomes. Fifteen ophthalmic surgeons from 11 public secondary and tertiary eye care centres in five regional Ethiopian states collected pre-intervention data using the BOOST app. Based on 20 consecutive patients with vision < 6/60, BOOST identified case selection, intraoperative complications or postoperative refractive error as the leading causes of poor outcomes. Each facility then invested 2500 United States dollars (US$) to target its leading cause, recorded outcomes for 60 consecutive cataract patients, and then compared the proportion with uncorrected vision ≥ 6/12 before and after the intervention.

**Local setting:**

All 11 centres are Orbis International partners and services provided at the facilities are highly subsidized or free of charge.

**Relevant changes:**

After implementing targeted interventions at the facilities to address undetected ocular comorbidity, surgical complications or postoperative refractive errors, 501 (56.0%) of 894 patients had good results (≥ 6/12), compared to 289 (32.2%) of 897 patients before the intervention.

**Lessons learnt:**

Using the BOOST app as an approach for quality monitoring and improvement enables benchmarking across contexts and can enhance surgical outcomes when paired with recommended targeted investments.

## Résumé

**Problème:**

L’Organisation mondiale de la Santé recommande une référence d’acuité visuelle non corrigée ≥ 6/12 chez 80% des patients, 4 à 6 semaines après une opération de la cataracte. Dans les pays à faibles revenus, il est difficile d’atteindre cette référence et d’évaluer les progrès réalisés.

**Approche:**

Cette intervention a nécessité des investissements modestes recommandés par l’application Better Operative Outcome Software Tool (BOOST). Ces investissements visaient des causes spécifiques de mauvais résultats à l’issue d’une opération de la cataracte dans les hôpitaux éthiopiens afin d’améliorer les résultats sur le plan de la vision. Quinze chirurgiens ophtalmologistes issus de 11 centres ophtalmologiques publics de niveau secondaire et tertiaire, répartis dans cinq États régionaux éthiopiens, ont recueilli des données pré-intervention à l’aide de l’application BOOST. Sur la base d’une étude portant sur 20 patients consécutifs présentant une acuité visuelle < 6/60, le programme BOOST a identifié la sélection des cas, les complications peropératoires ou l’erreur de réfraction postopératoire comme les principales causes de mauvais résultats. Chaque établissement a ensuite investi 2 500 dollars américains pour s’attaquer à la cause principale identifiée, a enregistré les résultats obtenus chez 60 patients consécutifs atteints de la cataracte, puis a comparé la proportion de patients présentant une acuité visuelle non corrigée ≥ 6/12 avant et après l’intervention.

**Environnement local:**

Les 11 centres sont tous partenaires de l’ONG Orbis International et les services fournis dans ces établissements sont fortement subventionnés ou gratuits.

**Changements significatifs:**

Après la mise en œuvre d’interventions ciblées dans les établissements pour traiter les comorbidités oculaires non détectées, les complications chirurgicales ou les erreurs de réfraction postopératoires, 501 (56,0%) des 894 patients ont obtenu de bons résultats (≥ 6/12), contre 289 (32,2%) des 897 patients avant l’intervention.

**Leçons tirées:**

L’utilisation de l’application BOOST comme outil de suivi et d’amélioration de la qualité permet une comparaison entre différents contextes et permet d’améliorer les résultats chirurgicaux lorsqu’elle est associée à des investissements ciblés recommandés.

## Resumen

**Situación:**

La Organización Mundial de la Salud recomienda como referencia una agudeza visual no corregida de ≥6/12 en el 80% de los pacientes entre 4 y 6 semanas después de la cirugía de cataratas. En países con recursos limitados, alcanzar este valor de referencia resulta difícil y evaluar los progresos es complejo.

**Enfoque:**

Esta intervención implicó inversiones modestas recomendadas por la aplicación Better Operative Outcome Software Tool (BOOST). Las inversiones se dirigieron a causas específicas de resultados deficientes de la cirugía de cataratas en hospitales de Etiopía, con el fin de mejorar los resultados visuales. Quince cirujanos oftalmólogos de 11 centros públicos de atención oftalmológica de nivel secundario y terciario en cinco estados regionales de Etiopía recopilaron datos previos a la intervención utilizando la aplicación BOOST. A partir de 20 pacientes consecutivos con una agudeza visual <6/60, BOOST identificó la selección de casos, las complicaciones intraoperatorias o el error refractivo posoperatorio como las principales causas de resultados deficientes. Cada establecimiento invirtió posteriormente US$ 2500 para abordar su principal causa, registró los resultados de 60 pacientes consecutivos con cataratas y comparó la proporción con agudeza visual no corregida ≥6/12 antes y después de la intervención.

**Marco regional:**

Los 11 centros son socios de Orbis International y los servicios prestados en estos establecimientos están altamente subvencionados o son gratuitos.

**Cambios importantes:**

Tras la implementación de intervenciones específicas en los establecimientos para abordar comorbilidades oculares no detectadas, complicaciones quirúrgicas o errores refractivos posoperatorios, 501 (56,0%) de 894 pacientes presentaron buenos resultados (≥6/12), en comparación con 289 (32,2%) de 897 pacientes antes de la intervención.

**Lecciones aprendidas:**

El uso de la aplicación BOOST como herramienta para el seguimiento y la mejora de la calidad permite la comparación entre contextos y puede mejorar los resultados quirúrgicos cuando se combina con inversiones específicas recomendadas.

## ملخص

**المشكلة:**

توصي منظمة الصحة العالمية بمعيار لمقارنة الحدة البصرية غير المصححة ≥ 6/12 لدى %80 من المرضى، بعد 4 إلى 6 أسابيع من جراحة الكتاراكت. في الدول ذات الموارد المحدودة، يُعد الوصول إلى هذا المعيار بمثابة التحدي، كما أن تقييم التقدم المتحقق أمر صعب.

**الأسلوب:**

تضمن هذا التدخل استثمارات متواضعة موصى بها من جانب تطبيق "الأداة البرمجية لتحقيق نتائج أفضل للعمليات الجراحية" (BOOST). استهدفت هذه الاستثمارات أسبابًا محددة لضعف نتائج جراحة الكتاراكت في مستشفيات إثيوبيا لتحسين نتائج الرؤية. قام 15 جراحًا للعيون من 11 مركزًا حكوميًا ثانويًا وثالثيًا للرعاية الصحية للعيون، في خمس ولايات إثيوبية إقليمية، قاموا بجمع بيانات ما قبل التدخل باستخدام تطبيق BOOST. استنادًا إلى بيانات 20 مريضًا متتاليًا يعانون في الرؤية < 6/60، فإن تطبيق BOOST قد حدد اختيار الحالات، أو المضاعفات أثناء العملية، أو أخطاء الانكسار بعد العملية، كأسباب رئيسية لضعف النتائج. قامت كل منشأة بعد ذلك باستثمار 2500 دولار أمريكي لاستهداف السبب الرئيسي، وسجلت النتائج لعدد 60 مريضًا متتاليًا خضعوا لجراحة الكتاراكت، ثم قارنت نسبة المرضى مع نظرائهم الذين لديهم رؤية غير مصححة ≥ 6/12 قبل وبعد التدخل.

**المواقع المحلية:**

جميع المراكز الأحد عشر هم شركاء مع منظمة Orbis International، والخدمات المقدمة لديها مدعومة بشكل كبير أو مجانية.

**التغيّرات ذات الصلة:**

بعد تطبيق تدخلات موجهة في المراكز لمعالجة أمراض العين المصاحبة غير المكتشفة، أو المضاعفات الجراحية، أو أخطاء الانكسار بعد الجراحة، حقق 501 مريضًا (%56.0) من أصل 894 مريضًا نتائج جيدة (≥ 6/12)¡ مقارنةً بـ 289 مريضًا (%32.2) من أصل 897 مريضًا قبل التدخل.

**الدروس المستفادة:**

إن استخدام تطبيق BOOST كأسلوب لمراقبة الجودة وتحسينها، يتيح إمكانية المقارنة المعيارية بين مختلف الأوضاع، ويمكنه تحسين نتائج الجراحة عند دمجها مع الاستثمارات الموجهة الموصى بها.

## 摘要

**问题:**

世卫组织建议了一项视力基准，在接受白内障手术后的 4 至 6 周，80% 的患者的裸眼视力应达到 ≥ 6/12。对资源匮乏国家而言，达到这一基准水平是一项极具挑战性的任务，且进度评估工作难以开展。

**方法:**

根据手术预后优化软件工具 (BOOST) 应用程序的建议，投入了金额适中的资金来实施该项干预措施。此类投入针对性排查导致埃塞俄比亚各医院白内障手术效果欠佳的具体原因，从而改善患者视力预后。来自埃塞俄比亚五个州级行政区 11 家公立二级和三级眼保健中心的十五名眼科手术医师运用 BOOST 应用程序收集了实施干预前基线数据。基于连续纳入 20 位视力 < 6/60 的患者，BOOST 确定，案例选择、术中并发症或术后屈光不正是导致手术结局欠佳的主要原因。之后每家医疗机构都投入了 2500 美元以找出导致其手术结局欠佳的主要原因，记录了 60 位连续白内障患者的手术预后数据，并比较了实施干预措施前后裸眼视力 ≥ 6/12 的患者所占比例。

**当地状况:**

全部 11 家中心均为国际奥比斯组织的合作伙伴，且此类医疗机构所提供服务均享有高额补贴或完全免费。

**相关变化:**

在此类医疗机构实施旨在解决未被发现的眼部合并症、手术并发症或术后屈光不正的针对性干预措施之后，894 位患者中有 501 位（占 56.0%）实现了良好的手术结果（裸眼视力达到 ≥ 6/12）；而在实施干预措施之前，897 位患者中仅 289 位（占 32.2%）获得了良好预后。

**经验教训:**

运用 BOOST 应用程序作为一种质量监测和改进方法，可实现不同情景的基准评估；若配合建议的针对性投入，则可提升手术预后。

## Резюме

**Проблема:**

Всемирная организация здравоохранения рекомендует в качестве целевого показателя достижение некорригированной остроты зрения на уровне ≥ 6/12 у 80% пациентов через 4–6 недель после операции по поводу катаракты. В странах с низким уровнем ресурсов достижение этого показателя является сложной задачей и оценка прогресса затруднена.

**Подход:**

Данное вмешательство включало умеренные инвестиции, рекомендованные программным приложением (программой) Better Operative Outcome Software Tool (BOOST). Инвестиции были направлены на конкретные причины неудовлетворительных результатов операций по поводу катаракты в больницах Эфиопии с целью улучшения результатов по зрению. Пятнадцать офтальмохирургов из 11 государственных офтальмологических центров вторичного и третичного звена в пяти регионах Эфиопии собрали доинтервенционные данные с использованием программы BOOST. На основе данных последовательной выборки по 20 пациентам со зрением < 6/60 программа BOOST определила в качестве основных причин неудовлетворительных исходов отбор пациентов, внутриоперационные осложнения и послеоперационные рефракционные ошибки. Каждое учреждение инвестировало 2500 долларов США в устранение основной причины, после чего были собраны данные последовательной выборки по 60 пациентам и выполнено сравнение доли пациентов с некорригированным зрением ≥ 6/12 до и после вмешательства.

**Местные условия:**

Все 11 центров являются партнерами организации Orbis International, а предоставляемые услуги в значительной степени субсидируются или оказываются бесплатно.

**Осуществленные перемены:**

После осуществления в учреждениях целенаправленных мероприятий для устранения недиагностированной сопутствующей патологии глаз, хирургических осложнений или послеоперационных рефракционных ошибок у 501 из 894 пациентов (56,0%) наблюдались хорошие результаты (≥ 6/12) по сравнению с 289 из 897 (32,2%) до вмешательства.

**Выводы:**

Использование программы BOOST как инструмента мониторинга и повышения качества позволяет сравнивать между собой различные условия и может улучшать исходы хирургических вмешательств при сочетании с рекомендованными целенаправленными инвестициями.

## Introduction

Cataract is the leading cause of vision impairment worldwide, affecting approximately 100.5 million people,^1^ yet it can be treated with simple, inexpensive surgery, restoring vision in 90% of patients.^2^ However, outcomes are often poor in low-resource settings,^3^ due to inappropriate case selection, surgical complications, uncorrected refractive errors and postoperative sequelae.^4^ Improving poor surgical outcomes is crucial to enhancing cataract surgical quality.^5^ The World Health Organization (WHO) proposes a benchmark for uncorrected visual acuity of ≥ 6/12 at 4 to 6 weeks following surgery in 80% of patients.^6^^,^^7^ However, this benchmark is challenging to attain and progress is difficult to assess in low-resource countries with poor postoperative follow-up. The difficulties are due to insufficient data collection, inadequate transportation infrastructure that restricts patients' ability to attend return visits, financial constraints and limited patient understanding of their disease.

A strategy for improving cataract surgical outcomes involves strengthening quality improvement processes within surgical centres.^8^ One of the challenges of collecting quality improvement data in low-resource settings can be a lack of tools and data management systems at the point of care. Recognizing this gap, stakeholders, including the nongovernmental organization Orbis International, developed the Cataract BOOST (Better Operative Outcomes Software Tool) application (app). The results of the Prospective Review of Early Cataract Outcomes and Grading (PRECOG) study informed the development of the app and created an international database that the BOOST app can access. BOOST can be downloaded onto Android devices and Windows computers and is free to use. The app allows users, in this case the cataract surgeons, to collect data immediately after surgery, when patient follow-up is highest;^8^ compare performance anonymously against other users locally and globally; and receive tailored suggestions addressing their most common causes of poor outcomes.

An initial study in 18 low- and middle-income countries found that BOOST facilitated better monitoring of outcomes, but despite users receiving BOOST-generated recommendations, patients’ visual outcomes did not improve.^9^ We hypothesized that targeted investments to remediate identified shortcomings would enhance the app’s impact. The current intervention aimed to test the hypothesis that modest investments, directed to specific parts of the operative process by user-specific BOOST feedback, would increase the proportion of patients meeting WHO vision targets postoperatively.

## Local setting

All participating facilities are government-owned and all services provided at the facilities, including cataract surgeries, are highly subsidized or free of charge. In Ethiopia, cataract surgeries are performed by ophthalmic surgeons, either ophthalmologists or non-physician cataract surgeons. Manual, small-incision cataract surgery is the predominant surgical approach across all the facilities.

## Approach

The National Research Ethics Review Committee of Ethiopia and regional health bureaus’ institutional review boards approved the intervention. Practising cataract surgeons and ophthalmologists recruited patients from 1 January 2022 to 30 May 2024.

### Pre-intervention

We selected 11 Orbis-partner secondary and tertiary hospitals in five regional states in Ethiopia for participation due to their having limited access to outcomes monitoring tools. The authors recruited 15 surgeon participants from eleven eye care units (three tertiary and eight secondary centres); 10 were trained ophthalmologists and five were non-physician cataract surgeons. These surgeons represented all available surgeons at the selected Orbis-partner hospitals. Participants downloaded the BOOST app onto mobile devices and Orbis Ethiopia personnel provided instruction on how to use the app for collecting cataract surgical data.^10^^,^^11^ To provide baseline data for comparison, before receiving and implementing targeted investments, each participating surgeon used BOOST to record de-identified demographic and clinical patient characteristics, including uncorrected pre-operative visual acuity in both eyes. They recorded the characteristics of 60 consecutive patients aged 35 years or older who had not previously undergone cataract surgery. Trained medical personnel (other than the surgeon) measured uncorrected visual acuity of the operative 1–3 days postoperatively, with surgeon identity masked at each facility.

After recruiting the 60 consecutive pre-intervention cataract patients, surgeon participants enrolled a further 20 consecutive postoperative cataract patients with poor (< 6/60) uncorrected visual acuity persisting after 4 weeks. In the BOOST app, the surgeons selected a reason for poor outcomes from the following three options: undetected ocular comorbidity, surgical complications or postoperative refractive error. BOOST then calculated the most common cause for poor outcomes at each facility and suggested targeted interventions. 

### Intervention with targeted investments

In collaboration with the Ethiopian health ministry, Orbis provided each participating facility with 2500 United States dollars (US$) to support the interventions recommended by BOOST to address the most common causes of poor surgical outcomes. This investment was not sufficient to upgrade all aspects of the surgical process but could address the most pressing issues when accurately identified. The facility and intervention personnel decided together which recommended intervention would most likely address the identified reason for poor vision outcomes. 

For example, the BOOST app suggested steps to improve the accuracy of the pre-operative examination, including provision of indirect ophthalmoscopes and training in their use to decrease undetected ocular comorbidities. Orbis Ethiopia, in collaboration with the principal investigators, organized training sessions to address the BOOST app recommendations. A local cornea surgeon and Assistant Professor at Jimma University delivered two training sessions, one week each. The training underscored the significance of addressing the challenges identified by BOOST through careful case selection and improved ocular biometry. Orbis Ethiopia also enhanced the stock of available lenses. Additionally, Orbis Ethiopia delivered surgical training to reduce intraoperative complications. Following the recommendations, 10 facilities provided training to improve case selection and address surgical complications; three facilities provided biometry equipment and associated training to improve refractive error outcomes; and 11 facilities procured a range of intraocular lenses and surgical instruments identified as needed to reduce complications and improve outcomes.

### Post-intervention

Surgeons recorded post-intervention uncorrected visual acuity in BOOST 1–3 days after surgery in the 60 additional consecutive cataract patients described above. The surgeons then compared the proportion achieving the WHO benchmark of ≥ 6/12 in the operative eye with pre-intervention results.

We used linear regression with generalized estimating equations accounting for the correlation of outcomes within surgeons to compare mean postoperative visual acuity before and after the intervention. We then summarized causes of poor visual outcome for cohorts at each facility and across the study.

## Relevant changes

### User satisfaction

Among 15 participating surgeons, four (26.7%) had performed fewer than 500 lifetime surgeries and 11 (73.3%) had performed more than 500. When asked, all the 15 surgeons reported that BOOST improved their subjective surgical performance.

### Causes of poor outcomes

Among 300 patients (20 consecutive patients for each of 15 surgeons) with poor outcomes, the leading cause was inappropriate case selection (188; 62.7%), that is, the presence of serious ocular morbidity besides cataract (Table 1). Surgical complications accounted for 63 (21.0%) patients and refractive error 49 (16.3%) patients. The leading cause of poor outcomes across surgeons was case selection (14; 93.3%) with surgical complication as the leading cause for one surgeon (1; 6.7%).

**Table 1:** Most common causes of vision loss for 20 consecutive patients with postoperative vision < 6/60 for each of 15 surgeon participants, Ethiopia, 2022–2024

Cause of poor visual outcome	No. cataract patients (%) *n* = 300
Case selection (ocular comorbidity besides cataract)	188 (62.7%)
Refractive error	49 (16.3%)
Surgical complication	63 (21.0%)

### Pre- versus post-intervention vision results

Among 897 patients included in pre-intervention analysis, 828 (92.3%) were blind (best corrected visual acuity < 3/60) in the pre-operative eye. Among 894 surgeries performed after the intervention, 853 (95.4%, *P*-value: 0.007) were blind pre-operatively. Most patients from both groups underwent small incision cataract surgery (pre-intervention cohort 896; 99.9%; and post-intervention cohort 892; 99.8%), with intraocular lens implantation, (pre-intervention cohort 891; 99.3%; and post-intervention cohort 892; 99.8%). The proportion meeting WHO benchmarks increased to 56.0% (501/894) post-intervention, compared with 32.2% (289/897) pre-intervention.

Mean uncorrected postoperative visual acuity improved from 0.592 (Standard Deviation (SD): 0.428) LogMAR [Snellen equivalent 6/24] pre-intervention to 0.441 (SD: 0.357) LogMAR [Snellen equivalent 6/19] post-intervention (*P-*value: < 0.0001).

## Lessons learnt

By providing a modest investment to participating facilities to implement the BOOST-generated recommendations, the proportion of patients achieving the WHO benchmark of ≥ 6/12 in the immediate postoperative period increased (Box 1). This intervention, therefore, aligns with WHO’s goals of enhancing cataract surgical outcomes, as part of delivering high-quality, patient-centred eyecare.^7^^,^^12^

Box 1 **Summary of main lessons learnt**

- Implementing targeted investments can lead to a significant increase in the proportion of cataract patients achieving the WHO vision benchmark in the immediate post-operative period.
- Strategies in Ethiopia should focus on enhancing quality of pre-operative examinations, to address comorbidities as the leading cause of poor visual outcomes.
- Using the BOOST app, or similar digital health quality monitoring platform, is beneficial when integrated as part of a broader quality improvement initiative, rather than as a standalone monitoring tool.

While our partners in Ethiopia experienced an improvement in postoperative outcomes, we note that effective use of the BOOST app is best suited to programmes that can integrate targeted funding with timely action, and we recognize that there can be financial challenges in addressing the recommendations generated by BOOST. Specifically, we aimed to implement the intervention in Zambia as well as in Ethiopia; however, the five Orbis partner hospitals in Zambia experienced significant delays due to logistical issues procuring the targeted investments, which didn’t allow for effective comparison of the intervention.

Additional factors contributed to delays and setbacks during project implementation. First, disruptions due to the coronavirus 2019 pandemic and related control measures caused implementation delays. Second, study participants encountered unexpected issues with the BOOST app, including the inability to synchronize and upload data and the need to update the app to reflect the > 6/12 benchmark (which was previously > 6/18 in earlier versions of the app). We communicated with the app developers to resolve these technical issues, but it still caused delays. Third, security concerns in Ethiopia interfered with some participants' routine activities and training availability, requiring close monitoring of the situation and collaboration with partner hospitals to implement strategies to prioritize participant safety. Finally, some participants faced challenges in identifying 20 patients with poor outcomes, so we scheduled additional support visits and arranged joint virtual calls to assist with identifying patients that met the eligibility criteria.

While the investments of US$ 2500 applied by this intervention may be unaffordable in some low-resource settings, the failure of a prior BOOST study to improve cataract surgical outcomes,^9^ suggests these modest investments may be necessary to realize significant outcome improvements.

Additional studies support the effectiveness of routine surgical outcome monitoring as a quality improvement tool in low- and middle-income countries.^13^^–^^15^ Through outcomes monitoring with the BOOST app, selection of patients with comorbidities was the most common cause of poor postoperative visual outcomes. Improving recognition of pre-existing ocular comorbidities can be addressed by improving comprehensive pre-operative examinations through practical training and the provision of equipment such as indirect ophthalmoscopes, as done in this intervention. Future studies should test locally relevant interventions to better identify ocular comorbidities besides cataract, including retinal diseases and glaucoma.

This article highlights the potential of appropriate digital-tools to support quality improvement in cataract surgery in low-resource settings. Future work should focus on scaling the use of digital apps, ensuring access to modest, targeted funding for equipment and training, and testing strategies to improve pre-operative examinations with implementation of a randomized controlled trial to increase confidence in findings.

## References

[1] Vision Loss Expert Group of the Global Burden of Disease Study; GBD 2019 Blindness and Vision Impairment Collaborators. Global estimates on the number of people blind or visually impaired by cataract: a meta-analysis from 2000 to 2020. Eye (Lond). 2024;38(11):2156–72. 10.1038/s41433-024-02961-1 38461217 PMC11269584

[2] Powe NR, Schein OD, Gieser SC, Tielsch JM, Luthra R, Javitt J, et al.; Cataract Patient Outcome Research Team. Synthesis of the literature on visual acuity and complications following cataract extraction with intraocular lens implantation. Arch Ophthalmol. 1994 Feb;112(2):239–52. 10.1001/archopht.1994.01090140115033 8037792

[3] Zaidi FH, Corbett MC, Burton BJ, Bloom PA. Raising the benchmark for the 21st century–the 1000 cataract operations audit and survey: outcomes, consultant-supervised training and sourcing NHS choice. Br J Ophthalmol. 2007 Jun;91(6):731–6. 10.1136/bjo.2006.104216 17050577 PMC1955623

[4] Zijlmans BL, van Zijderveld R, Manzulli M, Garay-Aramburu G, Czapski P, Eter N, et al.; European CAT-Community Study Group. Global multi-site, prospective analysis of cataract surgery outcomes following ICHOM standards: the European CAT-Community. Graefes Arch Clin Exp Ophthalmol. 2021 Jul;259(7):1897–905. 10.1007/s00417-021-05181-5 33855602

[5] Yan W, Wang W, van Wijngaarden P, Mueller A, He M. Longitudinal changes in global cataract surgery rate inequality and associations with socioeconomic indices. Clin Exp Ophthalmol. 2019 May;47(4):453–60. 10.1111/ceo.13430 30362287

[6] Burton MJ, Ramke J, Marques AP, Bourne RRA, Congdon N, Jones I, et al. The Lancet Global Health Commission on Global Eye Health: vision beyond 2020. Lancet Glob Health. 2021 Apr;9(4):e489–551. 10.1016/S2214-109X(20)30488-5 33607016 PMC7966694

[7] Summary of recommendations for quality of care in cataract surgery management. Geneva: World Health Organization; 2026. Available from: https://iris.who.int/handle/10665/384865 [cited 2026 Apr 14].

[8] Congdon N, Yan X, Lansingh V, Sisay A, Müller A, Chan V, et al. Assessment of cataract surgical outcomes in settings where follow-up is poor: PRECOG, a multicentre observational study. Lancet Glob Health. 2013 Jul;1(1):e37–45. 10.1016/S2214-109X(13)70003-2 25103584

[9] McGuinness MB, Moo E, Varga B, Dodson S, Lansingh VC, Resnikoff S, et al. The Better Operative Outcomes Software Tool (BOOST) Prospective Study: improving the quality of cataract surgery outcomes in low-resource settings. Ophthalmic Epidemiol. 2025 Feb;32(1):76–86. 10.1080/09286586.2024.2336518 38635874

[10] Congdon N, Suburaman GB, Ravilla T, Varga B, Resnikoff S, McLeod J, et al. Transforming research results into useful tools for global health: BOOST. Lancet Glob Health. 2016 Feb;4(2):e96. 10.1016/S2214-109X(15)00267-3 26823227

[11] BOOST Cataract by Auroitech [app]. 2020. Available from: https://play.google.com/store/apps/details?id=org.hollows.boost_health&hl=en_US [cited 2026 Apr 14].

[12] World report on vision. World Health Organization. Geneva: World Health Organization; 2019. Available from https://apps.who.int/iris/handle/10665/328717 [cited 2026 Apr 14].

[13] Yorston D, Gichuhi S, Wood M, Foster A. Does prospective monitoring improve cataract surgery outcomes in Africa? Br J Ophthalmol. 2002 May;86(5):543–7. 10.1136/bjo.86.5.543 11973251 PMC1771115

[14] Limburg H, Foster A, Gilbert C, Johnson GJ, Kyndt M. Routine monitoring of visual outcome of cataract surgery. Part 1: development of an instrument. Br J Ophthalmol. 2005 Jan;89(1):45–9. 10.1136/bjo.2004.045351 15615745 PMC1772455

[15] Limburg H, Foster A, Gilbert C, Johnson GJ, Kyndt M, Myatt M. Routine monitoring of visual outcome of cataract surgery. Part 2: results from eight study centres. Br J Ophthalmol. 2005 Jan;89(1):50–2. 10.1136/bjo.2004.045369 15615746 PMC1772465

